# Supplementary material for: MetaRibo-Seq measures translation in microbiomes
Source: Nat Commun. 2020 Jun 29;11:3268. doi: 10.1038/s41467-020-17081-z (PMC7324362; doi:10.1038/s41467-020-17081-z)
Supplement: Supplementary file 10 — Supplementary Data 7 [file 41467_2020_17081_MOESM10_ESM.zip › File2/Confidence_High_Taxonomy/118371_out.krona.html]

Javascript must be enabled to view this page.

members
magnitude
magnitudeUnassigned
count
unassigned
taxon
rank

118371\_out

4


SRS047044\_contig\_number\_contig-100\_10824.10824SRS077730\_contig\_number\_contig-100\_10740.10740
2

2
2
superkingdom

1
phylum
1239

class
1
186801

186802
order
1

186803
1
family

genus
1
841

species

SRS098827\_contig\_number\_12428
1
360807

203691
phylum
1

203692
class
1

136
1
order

1643685
family
1

64895
1
genus

64897

SRS053214\_contig\_number\_323
species
1
